# A Real-Time RT-PCR Assay for Genotyping of Rotavirus 

**DOI:** 10.29252/ibj.24.6.394

**Published:** 2020-06-13

**Authors:** Seyed Dawood Mousavi-Nasab, Farzaneh Sabahi, Hooman Kaghazian, Mahdi Paryan, Siamak Mirab Samiee, Mostafa Ghaderi, Fatemeh Zali, Manoochehr Makvandi

**Affiliations:** 1 Department of Research and Development, Production and Research Complex, Pasteur Institute of Iran, Tehran, Iran;; 2 Department of Virology, Faculty of Medical Sciences, Tarbiat Modares University, Tehran, Iran;; 3 Reference Health Laboratories Research Microbiology, Karaj Branch, Islamic Azad University, Karaj, Iran;; 4 Department of Microbiology, Karaj Branch, Islamic Azad University, Karaj, Iran;; 5 Department of Clinical Biochemistry, Faculty of Medicine, Tehran University of Medical Sciences, Tehran, Iran;; 6 Infectious and Tropical Diseases Research Center, Health research Institute, Ahvaz Jundishapur University of Medical Sciences, Ahvaz, Iran

**Keywords:** Gastroenteritis, Genotype, Real-time polymerase chain reaction, Rotavirus

## Abstract

**Background:**

HRV is the causative agent of severe gastroenteritis in children and responsible for two million hospitalizations and more than a half-million deaths annually. Sequence characteristics of the gene segments encoding the VP7 and VP4 proteins are used for the genotype classification of rotavirus. A wide variety of molecular methods are available, mainly based on reverse transcription PCR for rapid, specific and sensitive genotyping of rotaviruses. This study describes an alternative real-time PCR assay for genotyping of rotavirus.

**Methods:**

The samples of stools studied in this research have been collected from patients referred to Children's Medical Centers, Tehran, Iran. Rotavirus detection and genotyping were performed using the RT-PCR and semi-nested RT-PCR, respectively. Samples were then genotyped with a new real-time PCR.

**Results:**

The real-time PCR was able to genotype all positive samples with a mean C_t _of 28.2. Besides, a concordance rate of 100% was detected between real-time PCR and semi-nested RT-PCR.

**Conclusion:**

In this study, the genotyping of rotavirus with real-time PCR showed that this method can provide several favorable features, including high sensitivity and specificity, and a wide dynamic range for rotavirus genotyping.

## INTRODUCTION

Human rotavirus is the most common cause of severe gastroenteritis in infants and young children under the age of five years worldwide, accounting for two million hospitalizations and more than a half-million death every year^[^^1^^,^^2^^]^. Rotavirus is known to be transmitted person to person by the fecal-oral route. In developing countries, its transmission occurs through contaminated water. Base on reports, rotavirus can spread from child to child via the contamination of hands^[^^3^^]^. Rotaviruses are naked double-strand RNA viruses with a segmented genome and are classified as a separate genus (rotavirus) within the family *Reoviridae*^[^^4^^]^. Rotavirus genome is composed of 11 double-strand RNA segments, which are surrounded by the inner capsid proteins, including VP1, VP2, and VP3^[^^5^^]^. VP6 proteins form the middle layer of the virus capsid, and VP4 and VP7 proteins constitute the outer layer^[^^6^^]^. The Rotaviruses are currently categorized into eight groups, A through H, according to the group- and subgroup-specific antigens located at the VP6 region^[^^7^^]^. Four serogroups of rotavirus, including A, B, C, and H, are recognized to be human pathogens. Rotavirus group A is responsible for more than 90% of all cases^[^^4^^]^. Sequence characteristics of the segments encoding the VP7 [G, glycoprotein] and VP4 [P, protease-sensitive] are used for serotype and genotype classification. To date, group A rotaviruses have been grouped into at least 27 G and 37 P genotypes based on the differences in their VP7 and VP4 gene sequences, respectively. Among them, newly 12 G and 15 P genotypes are thought to infect humans^[^^8^^,^^9^^]^. G1, G2, G4, G9, and G non-type were shown to be the most prevalent G type, while P [8], P [4], and P non-type were found to be the most frequent P type in Iran, respectively^[^^10^^-^^12^^]^. 

Rotavirus classification methods have evolved primarily from antibody-based assays to genetic characterization^[^^13^^]^. Sequencing and phylogenetic analyses are currently considered to be the gold standard methods for HRV genotyping^[^^7^^,^^14^^,^^15^^]^. 

Nowadays, a wide variety of molecular methods, including Southern blot, Northern blot, reverse line blot hybridization, PCR-ELISA, and RFLP, have been developed for rapid, specific and sensitive genotyping of rotaviruses^[^^16^^,^^17^^]^. Multiplex semi-nested RT-PCR has been the primary rotavirus genotyping method discriminated by gel electrophoresis based on the amplicon length. Nearly all studies carried out in Iran have used multiplex semi-nested RT-PCR for genotyping HRV. Using high-throughput real-time PCR-based genotyping for rotaviruses has substantially reduced the risk of cross-contamination, resulting in faster turn-around time and higher sensitivity as compared with the conventional multiplex semi-nested RT-PCR^[^^18^^,^^19^^]^.

The purpose of this study was to investigate the potential value of a real-time PCR method for simple and fast genotyping of HRVs using Iranian strains. In the present study, we attempted to perform the analysis of a TaqMan real-time PCR assay that, to the best of our knowledge, has not yet been used for typing of HRV infections in Iran.

## MATERIALS AND METHODS

**Sample collection and processing**

A total of 120 stool samples were obtained from children aged five years and younger with a primary diagnosis of acute non-bloody gastroenteritis who refereed to Children’s Medical Center in Tehran, Iran, from May 2013 to May 2014. Criteria for collecting these samples were the absence of leukocytes, red blood cells and pus in the stool. The stools stored at -70 °C after primary analysis^[^^4^^]^.

A 10% (w/v) suspension of each stool sample was prepared for RNA extraction. Briefly, one gram (pea-sized) or 100 μl of each stool sample was dissolved in 1000 µl of PBS and then centrifuged at 1500 ×g for 20 minutes. Rotavirus RNA was extracted from 100 µl of the filtered supernatant by a standard phenol–chlorform extraction method^[^^20^^]^.

**Reverse transcription**

The cDNA was synthesized with the RevertAid RT Reverse Transcription Kit (Thermo Fisher Scientific, USA) according to the manufacturer’s instructions. Briefly, reverse transcription was carried out in a final volume of 20 μl containing 4 μl of 5× reverse transcription buffer, 1 μl of 10 mM dNTPs, 1 μl of 0.2 U/μl random hexamer, 1 μl of 40 U/μl RNase inhibitor, 1 μl of 200 U/μl reverse transcriptase enzyme, 8 μl of diethyl pyrocarbonate (RNase-free water), and 4 μl of the extracted RNA. The tubes were incubated at 42 ºC for 1 hour. 

**RT-PCR for rotavirus detection**

PCR amplification was carried out in a final volume of 25 µl containing 2.5 μL of the 10× PCR buffer (CinnaGen, Iran), 1 μL of each 10 pmol/μL primer, 1 μL of 10 mM dNTPs (Fermentas UAB, Lituania), 1 μL of 500 μ/μl of Taq DNA polymerase (CinnaGen), 1.5 μL of 50 mM of MgCl_2 _(CinnaGen), 12 μL of H_2_O, and 4 µl of the cDNA template. Two primers (forward: 5′-GAC GGV GCR ACT ACA TGG T-3′ and reverse: 5′-GTC CAA TTC ATN CCT GGT G -3′) were used to amplify the VP6 fragment^[^^21^^]^. The PCR conditions were set under the following conditions: an initial denaturation at 95 °C for 5 min, 40 cycles, including denaturation at 94 °C for 1 min, annealing at 55 °C for 1 min, elongation at 72 °C for 1 min, and a final extension step at 72 °C for 10 min. Amplifications were performed using the Verity™ *96-well Thermal Cycler* (Applied Biosystems, Foster City, CA, USA).

**Multiplex semi-nested RT-PCR**

G and P typing of HRV-positive samples were obtained by multiplex semi-nested RT-PCR assays using both consensus and type-specific primers, as described previously^[^^20^^]^. The PCR products were analyzed on a 2% agarose gel and visualized using GelRed dye (GelRed^ΤΜ^ Nucleic Acid Gel Stain).

**Real-time PCR** **for rotavirus genotyping**

Recently, Kottaridi *et al.*^[^^21^^]^ developed two panels of real-time RT-PCR assays for the detection of G1-G4 and G9, P [4], and P [8]. In our study, based on multiplex semi-nested RT-PCR result, the genotype-specific primers and probes were adapted or modified^[^^16^^,^^21^^]^. Both probes and primers were evaluated separately for each genotype based on the most conserved region, using the Allele ID^®^ (PREMIER Biosoft International) and ClustalW tools. In some cases, degenerate nucleotides were designed to ensure the amplification of all HRV genotypes. The oligonucleotides primers and probes were synthesized by Macrogen (Macrogen, South Korea). The six primer pairs, along with six probes labeled with different fluorophores, were used to amplify and detect genotypes G1, G2, G9, P4, P8, and the internal control (RNase P)^[^^21^^,^^22^^]^. The details of primers, probes and fluorophore/quenchers are shown in Table 1. To facilitate the use of real-time PCR and to enhance the sensitivity of the assay, three real-time PCR panels were formulated in this study, in which the panel I was designed for the detection of G1 and G2 genotypes, the panel II for the detection of G9 type as well Rnase P as internal control assay, and panel III for the identification of P [4] and P [8] genotypes. 

Real-time PCR genotyping was carried out in a LightCycler™96 system (Roche, Basel, Switzerland) Kottaridi *et al.*^[^^21^^]^ reported various annealing temperatures [55–65 °C] with the final concentrations range of 200 to 600 nmol for primers and probes. The real-time PCR reactions were performed in a 25-µl final volume, containing 2 µl of the cDNA template, 400 nM of each primer, and 200 nM of each probe. The Ct value was then determined. The amplification was performed under the following thermal conditions: initial denaturation at 95 ºC for 5 min, 40 cycles of denaturation at 95 ºC for 15 s, annealing at 60 ºC for 30 s, elongation at 72 ºC for 30 s, and a final extension step at 72 °C for 5 min. Based on the Ct value, the optimal conditions were determined, first for a single reaction and then for a set of multiple panels targeting G1/G2, G9/RNase P, and P4/P8 types. 

**Evaluation of real-time PCR performance**

The PCR products from each genotype were purified using the QIAquick PCR Clean-up Kit (*QIAGEN*, Hilden, Germany) and then cloned into the pTZ57R/T vector using the InsTAclone PCR cloning kit (Thermo Fisher Scientific). The plasmid was extracted using the DNA-spin Plasmid DNA Purification Kit (iNtRON Biotechnology, South Korea). Plasmid concentration was determined by measuring UV absorbance at 260 nm. The plasmid was then diluted in a Tris-EDTA buffer. Relative sensitivity and lower limit of detection of the assay determine the base of tenfold serial dilutions of each plasmid.

To assess the specificity and possible false-positive detection by the genotype-specific primer-probe sets, the positive samples for human adenovirus, norovirus, sapovirus, *Escherichia coli*, campylobacter, Cryptosporidium, and *Giardia lamblia* were investigated in this study. Additionally, partial sequencing was performed for 12 out of the 28 specimens to verify the accuracy of HRV genotyping by real-time PCR. Analysis of sequencing was blasted to determine the nucleotide identity (NCBI database) and further analysis was performed using MEGA version *6*^[^^23^^]^.

**Statistical analysis**

Comparison test was used to assess the consistency of real-time PCR and multiplex semi-nested RT-PCR results. A p value of less than 0.05 was considered to be statistically significant.

**Table 1:** *Primers and probes used for rotavirus genotyping by real-time PCR*

**Genotype**	**Primer sequences**	**Probe sequences**	**Fluorophore/ quencher**
G1F	AGCTGATTTGATATTGAATGAATGG	TCCACTTATTYGATTCTCCCGATTGYT	FAM/BHQ1
G1R	CACAGTACAYGATGATCCCATTG		
			
G2F	ACATTTGAGATTGTTGCVTCGTCTG	AGTGCRTTCGGTCCACCAACTTGAA	HEX/BHQ1
G2R	TGGAACTGTYGTTGGATCAGCAG		
			
G9F	ACTTGATGTDACTACAAATACCTG	ATCTAACACATCTGAGCCACCGACTTG	HEX/BHQ1
G9R	TGTGGTGYAGTAGTTGGATCYG		
			
P4F	TGAYGAAATAGARCAGATTGGATC	AATCTCTCCGTGTCCCCAATYRACTG	FAM/BHQ1
P4R	CCATCTAAAAYTGGTTCCACTG		
			
P8F	TAGACGTACACTAACTTCTGATAC	CACCATGAAATGTCCATATTCTTCCACC	HEX/BHQ1
P8R	TTGARCTATCRGTAGTAGCC		
			
RNase PF	AGA TTT GGA CCT GCG AGC G	TTCTGA CCTGAA GGCTCT GCG CG	FAM/BHQ1
RNase PR	GAG CGG CTG TCT CCA CAA GT		

F, forward; R, reverse

**Ethical statement**

The above-mentioned sampling protocols were approved by Tarbiat Modares University, Tehran, Iran (ethical code: 52/5140). A verbal consent and interview were taken from either parents of the enrolled child prior collection of stool samples.

## RESULTS

In the present study, 28 (23.3%) patients were found to be positive for HRV infection by RT-PCR method based on VP6 gene of rotavirus. The G and P genotypes were detected in 28/120 patients by multiplex semi-nested RT-PCR. Overall, G1 (75%) was found to be the most dominant genotype, followed by G2 (14.3%), G9 (7.14%), and mixed G1/G2 (3.58%). Moreover, in terms of p genotypes, P8 (75%) and P4 (25%) were dominant. Surprisingly, the G4 and G8 genotypes were not detected. 

**Performance criteria for real-time PCR genotyping**

***Accuracy and specificity***

Accuracy of RNA extraction and amplification were confirmed by the production of a signal from the RNase P internal control. There were no false-positive or false-negative results among the patients with the expected VP7 and VP4 genotypes. Regarding the isolated HRVs, no cross-reactivity was observed for adenovirus, strovirus, norovirus GI, norovirus GII, sapovirus, *Escherichia coli*, campylobacter, cryptosporidium, and Giardia lamblia. The results obtained by real-time PCR genotyping and partial sequencing of the amplified product showed 100% concordance to the both assays.

***Correlation between real-time PCR and multiplex semi-nested RT-PCR***

Both real-time PCR and multiplex semi-nested RT-PCR were able to detect all the 28 (100%), and the mean C_t_ value was 28.2. 

***Limit of detection***

**Fig. 1:**
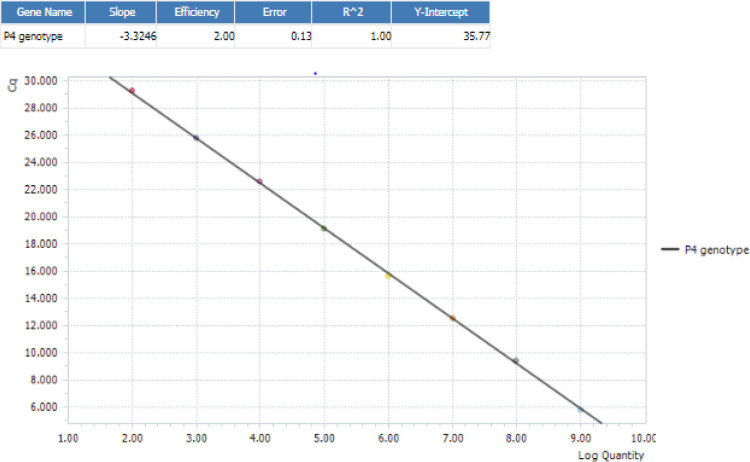
Limit of detection and real-time PCR performance for rotavirus P4 genotyping on LightCycler 96 system

To evaluate real-time PCR efficiency, standard curves were constructed using serial dilutions (from 10^-1 ^to 10^-10^) each plasmid. The lowest average of 10 copies per reaction could detect 100% of all known HVR genotypes, which have been identified in this study. The panel targeting the mixed G1/G2 genotypes yielded an r^2 ^≥ 0.94, while the panel targeting G9/RNase P and P4/P8 provided an r^2^ ≥ 0.99 for the standard curve (Table 2 and Fig. 1) 

## DISCUSSION

The severe gastroenteritis due to HRV occurs annually throughout the world, especially in developing countries, with high morbidity and mortality rates. The prevalence of different rotavirus genotypes varies in different geographical areas^[^^24^^,^^25^^]^. Interestingly, by using multiplex nested RT-PCR, the G1, G2, G9, mixed G1/G2, P4, and P8 genotypes were detected for VP7 and VP4; however, we did not detect some of the genotypes, such as G4 and G8^[^^10^^,^^11^^]^. The absence of these genotypes in our study is probably due to the geographical sampling bias. Although multiplex semi-nested RT-PCR assay has been recommended by WHO for HRV genotyping, this method is slow and prone to contamination. Moreover, the turn-around time for multiplex semi-nested RT-PCR assay is higher than real-time PCR assey^[^^20^^,^^21^^]^. The application of real-time PCR is generally regarded as a diagnostic tool since it provides quick, quantitative and reliable measurements. Furthermore, because real-time PCR-based genotyping methods were proved to be significantly cost-effective, this technique can serve as an alternative tool to the multiplex semi-nested RT-PCR method. Kottaridi *et al.*^[^^21^^]^ was applied the real-time PCR as a diagnostic tool for rotavirus genotyping, but in developing countries, because of genetic diversity and co-infection with other gastrointestinal pathogens, it is required to re-evaluate molecular techniques for the determination of rotavirus genotypes^[^^26^^,^^27^^]^. Therefore, these issues highlight the need for the analysis of a method that would allow the cost-effective, rapid and highly sensitive detection of HRV genotypes with a wider dynamic range. To the best of our knowledge, this is the first time that the real-time PCR assay has been utilized for the detection of HRV genotypes in Iran. For this purpose, the real-time PCR used to determine the G-P assay of rotavirus with TaqMan probes. 

The results of our TaqMan RT-PCR assay showed high accuracy and excellent correlation with conventional multiplex semi-nested RT-PCR and sequencing. The presented method also demonstrated high specificity and no cross-reaction with the above- mentioned pathogens. This assay with a lower limit of detection 10 copy/reaction was sensitive. Regrettably, we were not able to evaluate other HRV genotypes due to the limitation in the availability of samples in other geographical regions of Iran. 

**Table 2:** *Real-time PCR performance for rotavirus* *genotyping*

**Genotype ** **panel**	**r** ^2^	**Average efficiency (%) **
G1/G2	≥0.94	98
G9/RNase P	≥0.99	97
P4/P8	≥0.99	95

In conclusion, the rapid and cost-effective in-house real-time PCR assay evaluated herein for the HRV genotyping revealed high sensitivity and specificity, significantly shortened reaction time, and a wider dynamic range. 
